# Ultrasensitive detection of SARS-CoV-2 nucleocapsid protein using large gold nanoparticle-enhanced surface plasmon resonance

**DOI:** 10.1038/s41598-022-05036-x

**Published:** 2022-01-20

**Authors:** Taka-aki Yano, Taira Kajisa, Masayuki Ono, Yoshiya Miyasaka, Yuichi Hasegawa, Atsushi Saito, Kunihiro Otsuka, Ayuko Sakane, Takuya Sasaki, Koji Yasutomo, Rina Hamajima, Yuta Kanai, Takeshi Kobayashi, Yoshiharu Matsuura, Makoto Itonaga, Takeshi Yasui

**Affiliations:** 1 Institute of Post-LED Photonics, Tokushima University, 2-1 Minami-Josanjima, Tokushima, 770-8506 Japan; 2 JVCKENWOOD Corporation, 58-7, Shinmei-cho, Yokosuka, Kanagawa 239-8550 Japan; 3 Department of Immunology and Parasitology, Graduate School of Medical Sciences, Tokushima University, 3-18-15 Kuramoto, Tokushima, 770-8503 Japan; 4 Department of Biochemistry, Graduate School of Medical Sciences, Tokushima University, 3-18-15 Kuramoto, Tokushima, 770-8503 Japan; 5 Research Institute for Microbial Diseases, Osaka University, 3-1 Yamadaoka, Suita, Osaka 565-0871 Japan; 6 Center for Infectious Diseases Education and Research, Osaka University, 3-1 Yamadaoka, Suita, Osaka 565-0871 Japan; 7 Present Address: Graduate School of Interdisciplinary New Science, Toyo University, 2100, Kujirai, Kawagoe, Saitama 350-8585 Japan; 8 Present Address: Laboratory of Sericulture and Entomoresources, Graduate School of Bioagricultural Sciences, Nagoya University, Chikusa, Nagoya, 464-8601 Japan

**Keywords:** Imaging and sensing, Optical materials and structures

## Abstract

The COVID-19 pandemic has created urgent demand for rapid detection of the SARS-CoV-2 coronavirus. Herein, we report highly sensitive detection of SARS-CoV-2 nucleocapsid protein (N protein) using nanoparticle-enhanced surface plasmon resonance (SPR) techniques. A crucial plasmonic role in significantly enhancing the limit of detection (LOD) is revealed for exceptionally large gold nanoparticles (AuNPs) with diameters of hundreds of nm. SPR enhanced by these large nanoparticles lowered the LOD of SARS-CoV-2 N protein to 85 fM, resulting in the highest SPR detection sensitivity ever obtained for SARS-CoV-2 N protein.

## Introduction

The global pandemic of coronavirus disease (COVID-19) has continued without any sign of slowing down despite the significant increase in the numbers of vaccinated people^1^. The additional waves of the ongoing pandemic have created urgent demand for rapid, quantitative, on-site assays for detection of severe acute respiratory syndrome coronavirus 2 (SARS-CoV-2)^2–4^.

Molecular assays based on the reverse transcription polymerase chain reaction (RT-PCR) have been recognized as the gold standard for diagnostic testing of COVID-19^5,6^. However, the turnaround time for the diagnosis is long for the RT-PCR-based technique, as it requires sample transportation as well as sample processing associated with reverse transcription and amplification. In particular, RNA extraction remains a major bottleneck in viral RNA sampling from patient samples. Furthermore, this assay is also not amenable to onsite diagnosis.

As an alternative to RT-PCR assays via RNA detection, the detection methods of protein antigen for SARS-CoV-2 in sera and nasal secretions have been used with antibodies against nucleocapsid protein (N protein) and spike protein (S protein)^7,8^. Although these technique enables rapid diagnosis within around 15 min., they provide lower detection sensitivity than the RT-PCR technique, making it difficult to detect N protein as a standard method for the diagnosis. Hence, alternative techniques still need to be developed that can provide higher detection sensitivity along with rapid detection than conventional RT-PCR and N protein detection techniques.

Surface plasmon resonance (SPR) biosensors are a promising alternative for highly sensitive detection of SARS-CoV-2. They offer label-free and real time detection of antigen–antibody interactions at ultralow concentrations^9^. Qiu et al*.* used Au nanoparticles (AuNPs) functionalized with complementary DNA to demonstrate localized SPR (LSPR) sensing of oligonucleotides coding for SARS-CoV-2 sequences^10^. They combined the LSPR technique with a thermoplasmonic effect to achieve a sub-pM detection limit. Basso et al*.* succeeded in SPR detection of SARS-CoV-2 antibodies in serum from COVID-19-positive patients using Au SPR chips conjugated with functional self-assembled monolayers^11^. The effectiveness of SPR techniques notwithstanding, practitioners continue to demand improvements in SPR sensor limits of detection (LODs). A key breakthrough in the endeavor to transcend current LODs is the incorporation of AuNPs onto Au SPR chips^12^. AuNPs used in SPR sensors are usually conjugated with secondary antibodies in SPR measurements, thereby providing enhanced antigen–antibody binding affinity, analogous to that provided by a sandwich ELISA^13^. Furthermore, a strongly enhanced electromagnetic field is generated at the gap between AuNPs and the Au chip substrate due to the electromagnetic coupling between the LSPR in the AuNPs and the SPR on the planar Au chip substrate. The plasmonic coupling causes an SPR dip angle perturbation, which leads to significantly improved SPR sensitivity. Highly sensitive detections of a variety of biomedical analytes (proteins, aptamers, DNAs, RNAs, etc.) have already been demonstrated using nanoparticle-enhanced SPR^14–17^.

**Figure 1:**
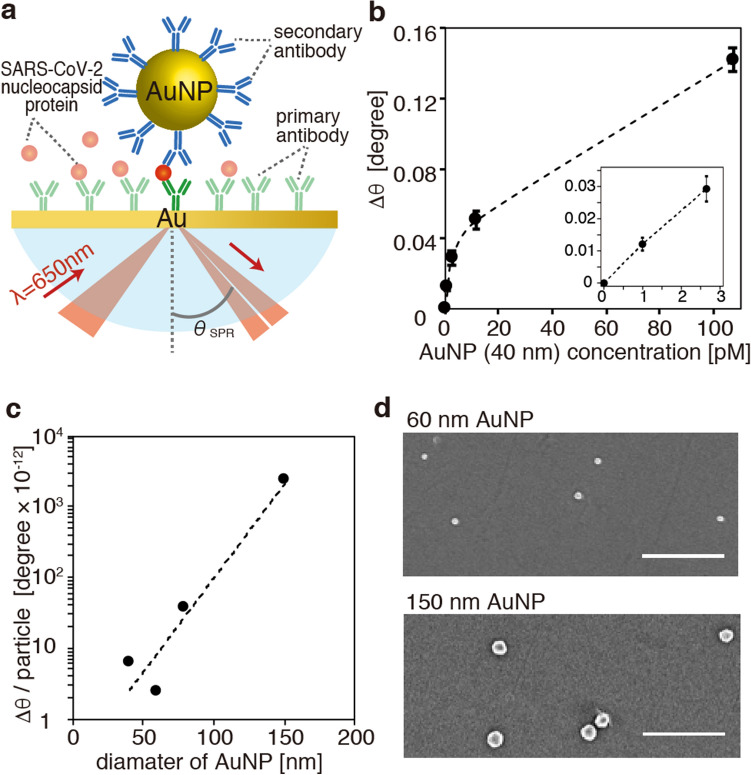
(**a**) Schematic of SPR measurement of SARS-CoV-2 nucleocapsid protein (N protein) using gold nanoparticles (AuNPs). (**b**) SPR angular shift (∆θ) as a function of concentration of AuNPs of 40 nm diameter. The inset shows an enlarged view in the low concertation region. (**c**) Angular shift induced by a single AuNP for AuNPs of various diameters. (**d**) SEM images of AuNPs of 60 nm and 150 nm diameters remaining on the gold-coated chip substrates after the SPR measurements in (**c**). The scale bars correspond to 1 µm.

Herein, we demonstrate nanoparticle-enhanced SPR sensing of SARS-CoV-2 N protein at fM levels using exceptionally large AuNPs with diameters of 150 nm, as schematically shown in Fig. 1a. The large AuNPs are found to exhibit SARS-CoV-2 detection sensitivity a full order of magnitude higher than the commonly-utilized smaller (tens of nanometers in diameter) AuNPs commonly utilized in nanoparticle-enhanced SPR. The crucial plasmonic roles of the large AuNPs in significant sensitivity enhancement are elucidated both experimentally and theoretically. Large nanoparticle-enhanced SPR is demonstrated to be more effective for large bioanalytes such as N protein.

## Results and discussion

Our SPR system was based on angular interrogation in the Kretschmann configuration with an Au film thickness of 50 nm and an excitation wavelength of 650 nm as detailed in Fig. S1. As AuNPs are expected to enhance SPR detection sensitivity, we first evaluated the dependence of the SPR angular shift (∆θ on AuNP concentration using avidin-conjugated AuNPs with a mean diameter of 40 nm. A HEPES buffer blank was initially incubated with a bare Au chip substrate for reference, and then ∆θ was measured in situ as the concentration of AuNP was increased stepwise every 10 min by replacing the AuNP-containing buffer solution. Figure 1b represents the value of ∆θ plotted as a function of AuNP concentration up to 100 pM. There are two linear regions observed in the plot with a larger slope in the very low-concentration region below 10 pM, as shown in the inset of Fig. 1b.

To evaluate the diameter dependence of the angular shift, we also measured ∆θ for AuNPs of a variety of diameters (60 nm, 80 nm, and 150 nm) in the low-concentration over which the linear relationship was observed. Taking the number of injected AuNPs into account, the amount of ∆θ induced by a single nanoparticle was estimated for each diameter. As shown in Fig. 1c, the single particle-induced ∆θ was found to increase significantly with increasing nanoparticle diameter. Figure 1d shows scanning electron microscope (SEM) images of AuNPs of 60 nm and 150 nm diameters remaining on the gold-coated chip substrates after the SPR measurements. Considering the numbers of injected AuNPs, the adsorption ratio of AuNPs on the chip substrates was estimated to be approximately 6%. The AuNP surface density at 10 pM shown in Fig. 1b, as deduced from the adsorption ratio, was 2.26 × 10^8^ particle/cm^2^. The nanoparticle density was thus approximately one order of magnitude lower than those previously reported^18,19^, validating the use of AuNPs at low-concentration. It should be noted that while high concentrations of AuNPs degrade the Q factor of the SPR curve, the low-concentration of AuNPs did not, as shown in Supplementary Fig. S2. The SPR dip angle can therefore be determined more accurately at low AuNP concentrations; this constitutes an additional advantage of using low AuNPs concentrations.

**Figure 2:**
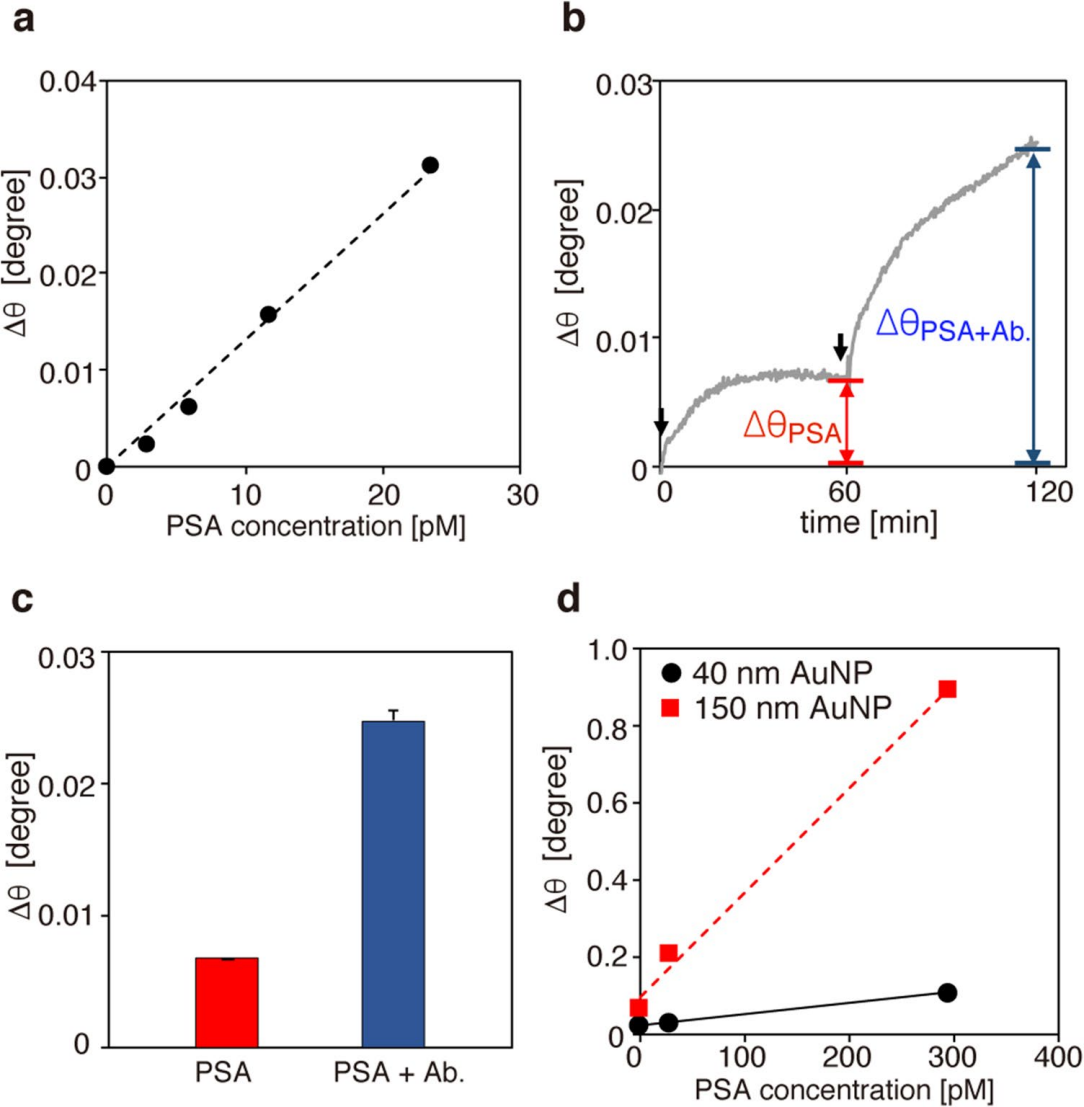
(**a**) SPR angular shift as a function of concentration of prostate-specific antigen **(**PSA) solution. (**b**) SPR sensorgram obtained after injection of 3 nM PSA solution at 0 min followed by injection of secondary anti-PSA antibody (Ab.) at 60 min. (**c**) SPR angular shift with and without the secondary antibody. (**d**) AuNP-enhanced SPR angular shifts as functions of PSA concentration for AuNPs of 40 nm and 150 nm diameters.

Inspired by the large nanoparticle-enhanced shift in ∆θ, we demonstrated highly sensitive detection of antigens using AuNPs of 150 nm diameter. Prostate-specific antigen (PSA) was utilized for this demonstration, since the size of PSA (34 kDa) is comparable to that of SARS-CoV-2 N protein (49.1 kDa). Initially, SPR detection of PSA antigens (3 nM) was performed using an anti-PSA antibody (1H12)-coated Au chip with the neither use of secondary antigens or AuNPs. Figure 2a shows the shift in the SPR angle (∆θ) measured as the PSA concentration changed from 0.3 nM (100 ng/ml) to 24 nM (800 ng/ml). The figure clearly exhibits a linear increase with increasing PSA concentration, resulting in a LOD of 2.78 nM. Figure 2b shows an SPR sensorgram obtained as the 3 nM PSA solution was first incubated for 60 min and incubated for a further 60 min with secondary anti-PSA antibody (5A6) at a concentration of 0.4 µg/ml. Although ∆θ reached a plateau 60 min after injection of PSA solution, additional injection of the secondary antibodies induced a significant increase in ∆θ owing to enhanced antigen–antibody affinity. As summarized in Fig. 2c, the sandwich assay using the secondary antibodies provided an angular interrogation shift 3.7 times as large. Finally, an AuNP-based sandwich assay was performed using secondary antibody-conjugated AuNPs. After incubation with PSA solution for 30 min, an AuNP solution with an optical density of 0.5 was injected, and the SPR angular shift was measured 60 min after the injection. Figure 2d shows the SPR angular shifts for AuNPs of 40 nm and 150 nm diameters, measured as functions of PSA concentration. Both AuNP sizes exhibited linear increases in SPR angular shift with increasing PSA concentration. Significantly, the AuNPs of 150 nm diameter provided a ∆θ value one full order of magnitude larger than that of the AuNPs of 40 nm diameter. Notably, the AuNPs of 150 nm diameter enhanced the angular shift by a factor of 120 even though the PSA concentration was ten times lower than that used in the AuNP-free SPR measurements in Fig. 2a. This angular shift enhancement resulted in an overall detection sensitivity enhancement by a factor of 1200.

**Figure 3:**
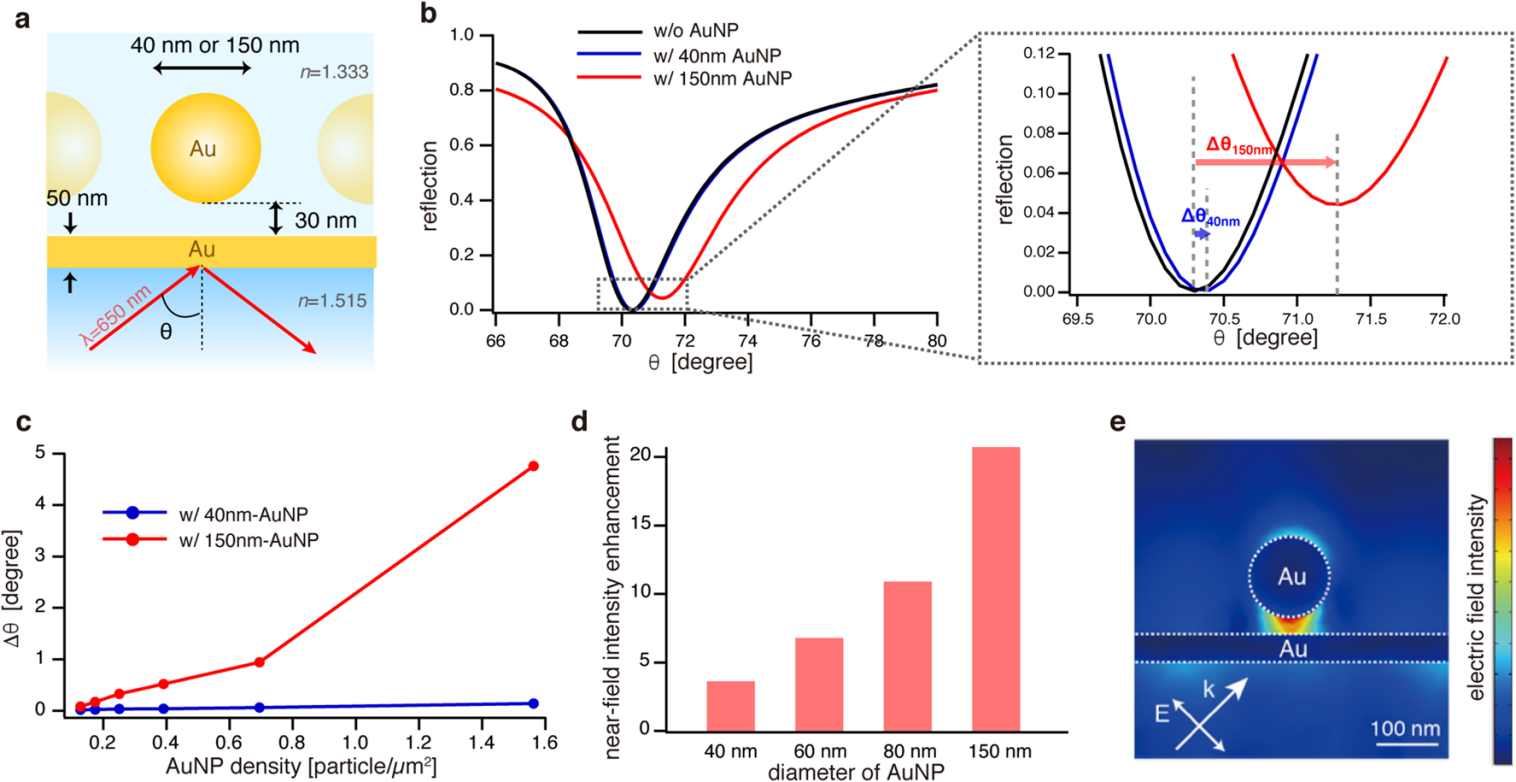
(**a**) Simulation model of nanoparticle-enhanced SPR. (**b**) Angle-resolved reflection spectra (SPR curves) with and without AuNPs of 40 nm and 150 nm diameters. (**c**) SPR angular shift as a function of AuNP density for AuNPs of 40 nm and 150 nm diameters. (**d**) Diameter-dependent near-field intensity enhancement at the gap between an AuNP and the Au chip substrate. (**e**) Electric field distribution in the vicinity of an AuNP of 150 nm diameter.

To investigate the plasmonic roles of the large AuNPs in the higher detection sensitivities of the antigens, we simulated the SPR curves for AuNPs of 40 nm and 150 nm diameters using finite element analysis (COMSOL Multiphysics 5.6). Figure 3a shows a simulation model in which AuNPs with diameters of 40 nm or 150 nm were placed on a 50 nm-thick Au film with a gap distance of 30 nm corresponding to the thickness of the antibody–antigen–antibody sandwich. The angle-resolved SPR spectra in Fig. 3b were obtained by calculating the reflection of the *p*-polarized incident light (λ: 650 nm) over a range of incident angles. The AuNP concentration was set to 0.69 particles per square micrometer. Compared with the AuNP-free case, the AuNPs of 40 nm diameter produced a 0.06 degree increase in the SPR angle, whereas AuNPs of 150 nm diameter produced a 0.94 degree increase. This finding indicates significant enhancement of SPR sensitivity by AuNPs of 150 nm diameter. Figure 3c shows SPR angular shifts plotted as functions of AuNP concentration for AuNP diameters of 40 nm and 150 nm. For AuNPs of 40 nm diameter, the angle shift increased almost linearly with increasing AuNP concentration. On the other hand, for AuNPs of 150 nm diameter, the shift was not linearly proportional to the concentration, but at low concentrations (< 0.07 particle/µm^2^), a linear increase in angular shift was obtained with increasing AuNP concentration. For AuNPs of 150 nm diameter, the slope at low concentrations was 1.5 degree/particle, which is 16 times higher than for AuNPs of 40 nm diameter. Therefore, AuNPs of 150 nm diameter provided detection sensitivity more than one order of magnitude higher than AuNPs of 40 nm diameter in SPR measurement; this finding is in good agreement with the experimental data in Fig. 2d.

This behavior is caused by efficient excitation of the gap-mode plasmon between AuNPs of 150 nm diameters and the Au thin film, significantly affecting the SPR signal. For the relatively large gap distance (~ 30 nm), AuNPs of 150 nm diameter are superior to those of 40 nm diameter in terms of both field enhancement and confinement. As the diameter of AuNPs increases, the resonant wavelength of the gap mode plasmon gets closer to the excitation wavelength (650 nm), resulting in the higher near-field intensity at the large gap for AuNPs of 150 nm as shown in Fig. 3d. Furthermore, in terms of field confinement for the large gap distance, AuNPs of 40 nm diameter confined incident light within a closer vicinity of the particle surface than AuNPs of 150 nm diameter, making it less efficient for the confined field to couple with the gold thin film at the large gap distance. Figure 3e shows the calculated electric field distribution in the vicinity of the AuNPs of 150 nm diameter and the Au thin film with the large gap distance of 30 nm at the incident wavelength of 650 nm. This depiction highlights the strongly localized field induced by the gap-mode plasmon at the gap. Such an efficient excitation of the gap-mode plasmon by AuNPs of 150 nm diameter contributed to SPR sensitivity enhancement.

**Figure 4:**
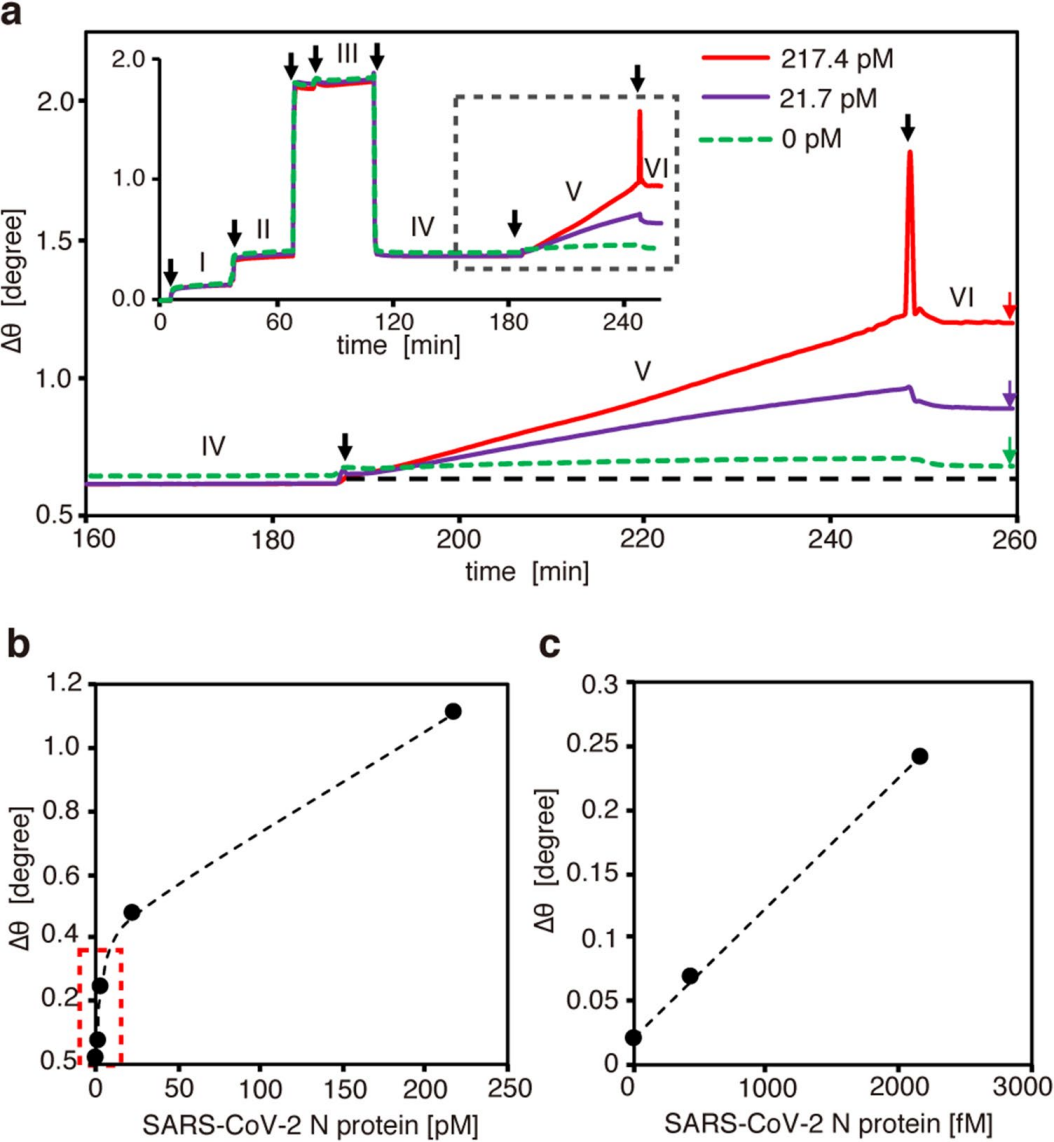
(**a**) Sensorgram of AuNP-enhanced SPR detection of SARS-CoV-2 N protein. (**b**) SPR angular shifts measured while increasing the concentration of SARS-CoV-2 N protein. (**c**) Enlarged view of the low-concentration region of (**b**).

Finally, the large gold nanoparticle-enhanced SPR was applied in the detection of SARS-CoV-2 N protein. Figure 4a shows a sensorgram that simultaneously recorded the whole detection process for each of three concentrations of N protein in a 3-well chamber. After baseline measurement with phosphate-buffered saline (PBS) buffer solution, 10 µg/mL of an anti-N protein antibody (B3451M) was introduced, followed by incubation for 30 min (Fig. 4a, solid line I). The SPR chip was then rinsed and incubated in PBS-T (PBS containing 0.05% Tween20; blocking buffer) for 30 min (Fig. 4a, solid line II). Immediately after rinsing with N protein-diluted buffer, N protein-diluted buffer solutions of three different concentrations (0 pM, 21.7 pM, and 217.4 pM) were simultaneously added to each well of the sample chamber, followed by incubation for 30 min (Fig. 4a, solid line III). After washing of the chip substrate in PBS-T (Fig. 4a, solid line IV), 150 nm AuNPs conjugated with secondary antibodies of biotinylated anti-N protein (B3449M) in PBS-T including 0.1% casein were added, followed by incubation for 1 h (Fig. 4a, solid line V). Finally, after washing of the substrate with PBS-T, the SPR dip angles for three concentrations were simultaneously monitored (Fig. 4a, solid line VI). As shown in the enlarged view of Fig. 4a, the SPR angles were sensitive to the N protein concentration, increasing with the addition of N protein. Furthermore, SPR measurements for lower N protein concentrations were also performed in the same manner, as summarized in Fig. 4b. The SPR angular shift ∆θ showed its highest sensitivity to N protein concentration at concentrations below 20 pM. Figure 4c shows an enlarged view of Fig. 4b, depicting the linear slope in the low-concentration region. Considering the angular detection limit (0.0022 degree) and its coefficient of variation (10%), the LOD is estimated to be 85 fM (4 pg/ml), resulting in the highest SPR detection sensitivity ever obtained for SARS-CoV-2 N protein.

In conclusion, highly sensitive detection of SARS-CoV-2 N protein by SPR in a sandwich configuration was demonstrated using exceptionally large AuNPs of 150 nm diameter. AuNPs of 150 nm diameters were found to efficiently induce the gap-mode plasmon between the AuNPs and the Au chip substrate, leading to a tenfold enhancement of the SPR angular shift compared with AuNPs of 40 nm diameters. Furthermore, a low-concentration of AuNPs (< 10 pM) was probed and found to be effective in suppressing degradation of the Q factor in the SPR curves; this suppression contributed to highly accurate determination of the SPR angle. The large nanoparticle-enhanced SPR sensors via the use of the large AuNPs with diameters of hundreds of nm is a promising technique for sensing a variety of bioanalytes with sizes of several tens of nm.

Although the LOD (85 fM; 4 pg/ml) of N protein achieved by our SPR-based technique is comparable to analytical sensitivity of typical RT-PCR testing (LOD: ~ 30 copies/ml; 4.5 pg/ml), the SPR technique does not suffer from long time for sample preparation in comparison with RT-PCR, enabling to absolutely reduce the turnaround time for sample-to-result. The SPR-based system is constructed by assembling simple optical components with an inexpensive camera and LED light source, making the diagnostic device available at a price of < $10,000. Furthermore, the SPR-based technique does not require to educate technicians for diagnosis while PCR-based diagnosis needs to train technicians for extraction of RNA from patient specimens, which accelerates practical implementation. Owing to the highly-sensitive, cost-effective and easy-to-handle capabilities, the novel SPR-based technique is a promising alternative to currently available diagnostic tools, and would have a great potential to not only detect but also identify SARS-CoV-2 variants if it is combined with plasmon-enhanced vibrational spectroscopies.

## Materials and methods

### Reagents and chemicals

All the chemicals and reagents used were of analytical grade. The SARS-CoV-2 N protein antibodies used were mouse monoclonal antibodies purchased from Meridian Life Science (B3451M and B3449M). Prostate-specific antigen (PSA) and anti-PSA antibodies (5A6 and 1H12) were purchased from HyTest. Au nanoparticles with diameters of 40, 60, 80, and 150 nm were purchased from Sigma-Aldrich (753637, 753653, 753661, and 746649, respectively). Blocking reagent (Blocker Casein in PBS 37582) was purchased from Thermo Fisher Scientific. Neutralized avidin from egg white was purchased from FUJIFILM Wako Pure Chemical Corp.

### Multichannel surface plasmon resonance system

The SPR system used was developed by modifying a customizable SPR unit (Kyushu Keisokki Co., Ltd., Japan). As schematically shown in Supplementary Fig. S1a, it was based on angular interrogation in the Kretschmann configuration consisting of a hemicylindrical glass prism in contact with a gold-coated glass slide (10 mm × 10 mm). For biohazard protection, a disposable polystyrene cuvette was employed for analyte injection and was immobilized on the gold-coated glass slide using a compressive latch. The plastic cuvette possessed three wells with each maximum injection volume of 70 µl, providing parallel SPR measurements of three samples of different concentrations. In each measurement, 50 µl of analyte was injected into each well. The entire SPR system was mounted inside a thermally insulating box. The temperature inside the box was maintained at 25 °C or 37 °C by means of Peltier elements in order to suppress thermally induced drift of the SPR curves.

As for the optical configuration, the *p*-polarized component of an LED light with wavelength 650 nm was incident to the prism with angular convergence generated by a cylindrical lens. The reflected light with angular diffusion was captured by a CMOS camera through relay lenses, enabling the camera to detect the reflected light with incident angles ranging from 65° to 73° with angular resolution of 0.00001°. With the use of custom-made software, user-selected regions of interest were defined for each well on the camera image, providing the angle-resolved SPR curve for each well. The SPR angle for each well was determined from the dip position with minimum intensity in the SPR curve and was monitored in real time.

All the solutions used were preheated to the working temperature of 25 °C or 37 °C. In all experiments, the shift in SPR angle (∆θ was determined from the change in the SPR angle averaged over a period from 1 min before to 1 min after analyte injection.

### Immunoassays

There were three types of immunoassays demonstrated in this study, as illustrated in Supplementary Fig. S1b. One (Fig. S1b (i)) was a non-labeling immunoassay in which injected antigens were captured by monoclonal antibodies immobilized on the gold-coated substrate. The other two immunoassays were sandwich assays with the use of labeled secondary antibodies. In one case (Fig. S1b (ii)), biotinylated secondary antibodies were injected into the medium and incubated with antigens. In the other case (Fig. S1b (iii)), avidin-conjugated Au nanoparticles were further injected along with the biotinylated secondary antibodies. The biotinylated antibodies were likely to be assembled to avidin-coated Au nanoparticles due to the high affinity between biotin and avidin. The antibody-conjugated Au nanoparticles were finally bound to the antibody-immobilized Au surface together with antigens as a sandwich.

### Preparation of Au-coated chip substrate

Sputtering was used to coat the chip substrates (1 mm-thick BK7) with 3 nm of Cr as an adhesive layer, followed by 50 nm of Au. A process chamber was first pumped down to 4.0 × 10^−4^ Pa, followed by introduction of argon gas at a flow rate of 8.3 × 10^−7^ m^3^/s and a pressure of 0.48 Pa for the sputtering.

### Preparation of biotinylated antibodies

Antibodies were biotinylated using a Biotin-Labeling Kit-NH2 (Dojindo Laboratories) according to the manufacturer’s instructions.

### Preparation of avidin-conjugated Au nanoparticles

Au nanoparticles were incubated with 5.0 mg/mL neutravidin in 10 mmol/L carbonate–bicarbonate buffer (pH 9.75) for 2 h at room temperature. Then, 1% casein in PBS was added, followed by incubation for 1 h at room temperature. After removing unbound avidin and casein, the buffer was replaced with 200 μL of 4-(2-hydroxyethyl)-1-piperazineethanesulfonic acid (HEPES) buffer (10 mmol/L HEPES; pH 7.9) containing 50 mmol/L KCl, 1.0 mmol/L ethylenediaminetetraacetic acid (EDTA), and 0.1% polysorbate 20. The mixture was then stored at 4 °C until use.

### Imaging of Au nanoparticles

The Au-coated chips were coated with 1.5 nm of platinum by vacuum deposition to provide conductivity and were then subjected to SEM analysis. Secondary electron images were acquired by a field emission SEM (S-5200, Hitachi, Ltd.) at an accelerating voltage of 3 kV.

### Estimation of the number of Au nanoparticles adsorbed on Au substrates

The number of Au nanoparticles adsorbed on Au substrates was statistically counted from the SEM images. The adsorption ratio was calculated based on injected amount of the nanoparticles.

### Recombinant virus construction

The cDNA of the SARS coronavirus-2 nucleocapsid (SARS-CoV-2-N) gene with N-terminal His-tag sequence, which was codon-optimized for *Spodoptera frugiperda*, was chemically synthesized (Invitrogen) and then cloned into baculovirus transfer vector pFastBac1 (Invitrogen), generating pFastBac-His-SARS-CoV-2-N(Sf) plasmid. The recombinant bacmid AcBac/His-SARS-CoV-2-N(Sf) was constructed through transposition with pFastBac-His-SARS-CoV-2-N(Sf) using a Bac-to-Bac baculovirus expression system (Invitrogen) in accordance with the manufacturer’s instructions. To amplify the recombinant baculovirus derived from AcBac/His-SARS-CoV-2-N(Sf), *Spodoptera frugiperda* Sf9 cells were transfected with AcBac/His-SARS-CoV-2-N(Sf) using Lipofectamine 2000 Transfection Reagent (Invitrogen). At 144 h post-transfection, the culture medium containing progeny viruses was collected and then used for further amplification of the recombinant baculoviruses.

### Recombinant protein purification

Sf9 cells were infected with AcBac/His-SARS-CoV-2-N(Sf) virus and harvested at 72 h post-infection. Cells were lysed in lysis buffer (20 mM Tris–HCl pH 8, 135 mM NaCl, 1% Triton, 10% Glycerol) and incubated on ice for 5 min. The cell lysates were then sonicated for five rounds of sonication cycles of 30 s ON and 30 s OFF at HIGH power setting using a Bioruptor II. The insoluble material was removed by centrifugation at 7,000 rpm for 15 min at 4 °C. The solubilized extract was then incubated with cOmplete His-Tag Purification Resin (Roche) for 16 h at 4 °C with rotation. The resin was harvested by centrifugation at 7,000 rpm for 3 min at 4 °C and washed five times with wash buffer (20 mM Tris–HCl pH 8, 135 mM NaCl, 1% Triton, 10 mM Imidazole). His-N protein was then eluted five times with elution buffer (20 mM Tris–HCl pH 8, 135 mM NaCl, 1% Triton, 250 mM Imidazole). The purified His-N protein was subjected to SDS-PAGE, followed by Coomassie Brilliant Blue (CBB) staining. As indicated by the arrowhead in Supplementary Fig. S3, the 49.1 kDa protein was detected as His-N protein. The full-length image of the gel is presented in Supplementary Fig. S4. The concentration of purified His-N protein was 600 µg/ml.

## Supplementary Information

Supplementary Information.
