# Supplementary material for: Ultrasensitive detection of SARS-CoV-2 nucleocapsid protein using large gold nanoparticle-enhanced surface plasmon resonance
Source: Sci Rep. 2022 Jan 20;12:1060. doi: 10.1038/s41598-022-05036-x (PMC8776812; doi:10.1038/s41598-022-05036-x)
Supplement: Supplementary file 1 — Supplementary Information. [file 41598_2022_5036_MOESM1_ESM.pdf]

Supporting Information

Ultrasensitive detection of SARS-CoV-2 nucleocapsid protein using large gold nanoparticle-enhanced surface plasmon resonance

Taka-aki Yano[1]*, Taira Kajisa[1], Masayuki Ono[2], Yoshiya Miyasaka[2], Yuichi Hasegawa[2], Atsushi Saito[2], Kunihiro Otsuka[1,3], Ayuko Sakane[1,4], Takuya Sasaki[4], Koji Yasutomo[1,3], Rina Hamajima[5], Yuta Kanai[5], Takeshi Kobayashi[5], Yoshiharu Matsuura[5,6], Makoto Itonaga[2], and Takeshi Yasui[1]*

[1]Institute of Post-LED Photonics, Tokushima University, 2-1 Minami-Josanjima, Tokushima 770-8506, Japan

[2]JVC KENWOOD Corporation 58-7, Shinmei-cho, Yokosuka, Kanagawa 239-8550, Japan

[3]Department of Immunology & Parasitology, Graduate School of Medical Sciences, Tokushima University, 3-18-15 Kuramoto, Tokushima 770-8503, Japan

[4]Department of Biochemistry, Graduate School of Medical Sciences, Tokushima University, 3-18-15 Kuramoto, Tokushima 770-8503, Japan

[5]Research Institute for Microbial Diseases, Osaka University, 3-1 Yamadaoka, Suita, Osaka 565-0871, Japan

[6]Center for Infectious Diseases Education and Research, Osaka University, 3-1 Yamadaoka, Suita, Osaka 565-0871, Japan

*Corresponding authors: yano.takaaki@tokushima-u.ac.jp, yasui.takeshi@tokushima-u.ac.jp

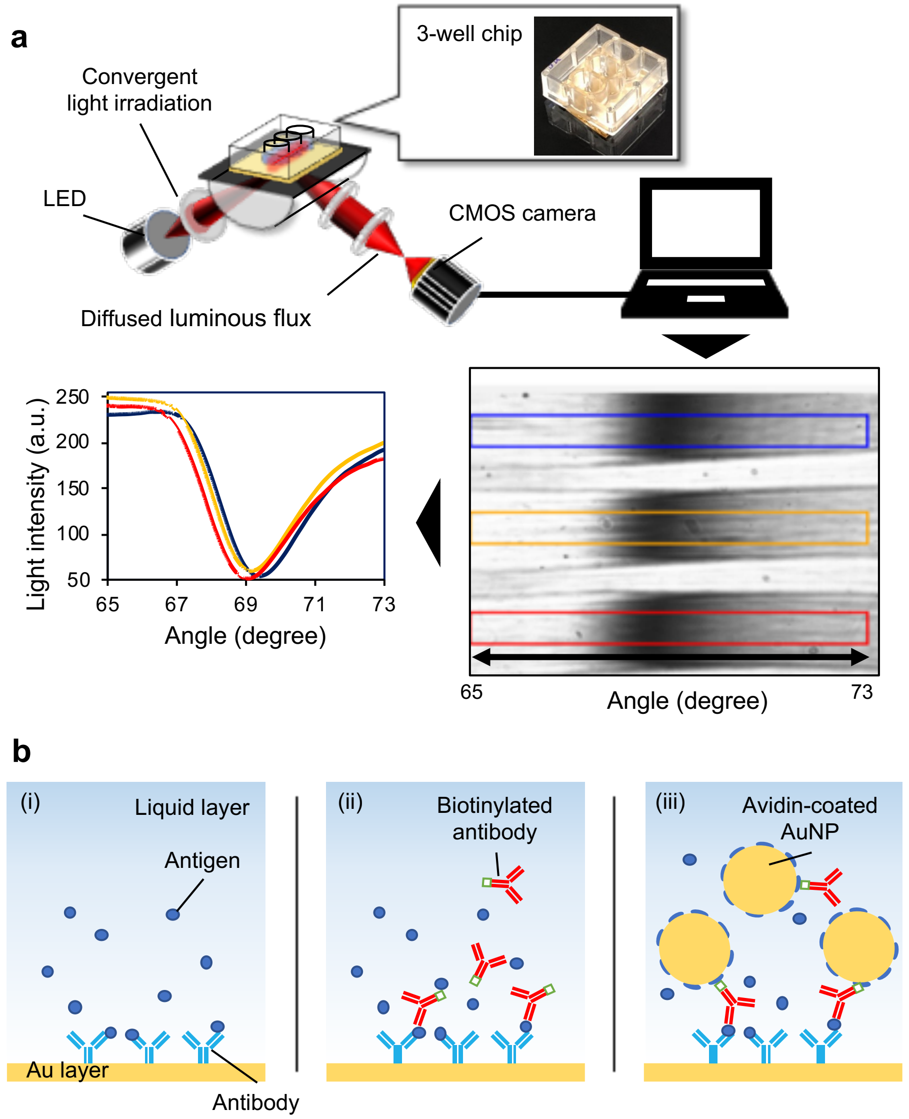

Figure S1: (a) schematic of a multichannel SPR system employing the Kretschmann configuration with p-polarized LED light (λ: 650 nm). A disposable 3-well cuvette is equipped for simultaneously obtaining multichannel SPR sensorgrams. (b) schematic of immunoassays with (i) and without (ii, iii) biotinylated secondary antibodies. Avidin-conjugated Au nanoparticles are also added in (iii).

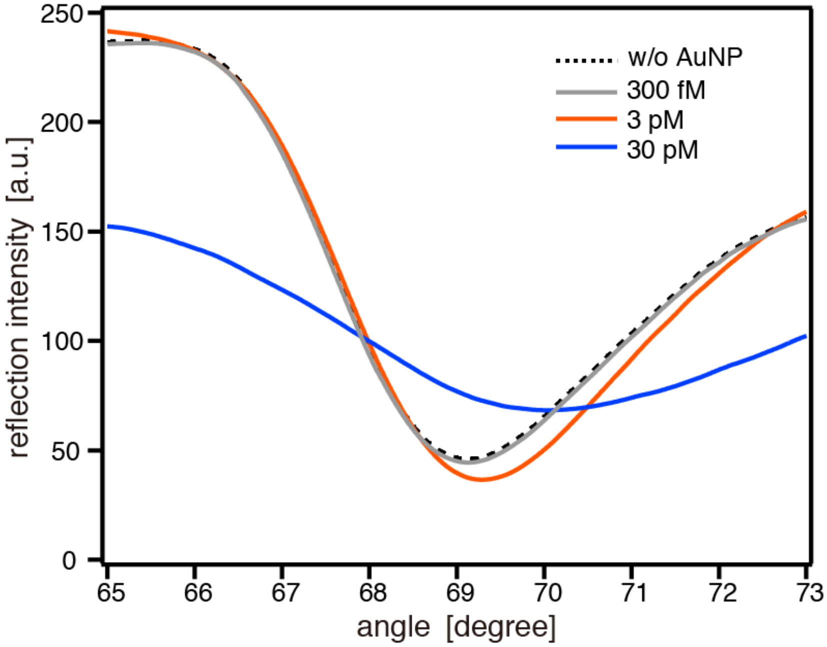

Figure S2: SPR curves measured after injecting 150 nm-AuNPs with different concentrations (300 fM, 3 pM and 30 pM).

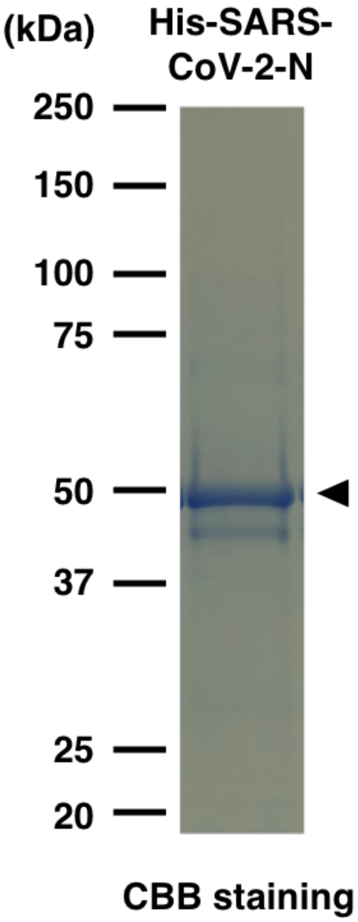

Figure S3: SDS-polyacrylamide gel electrophoresis (SDS-PAGE) of the purified His-tagged SARS-CoV-2 N protein, followed by Coomassie Brilliant Blue (CBB) staining. The uncropped gel is shown in Figure S4.

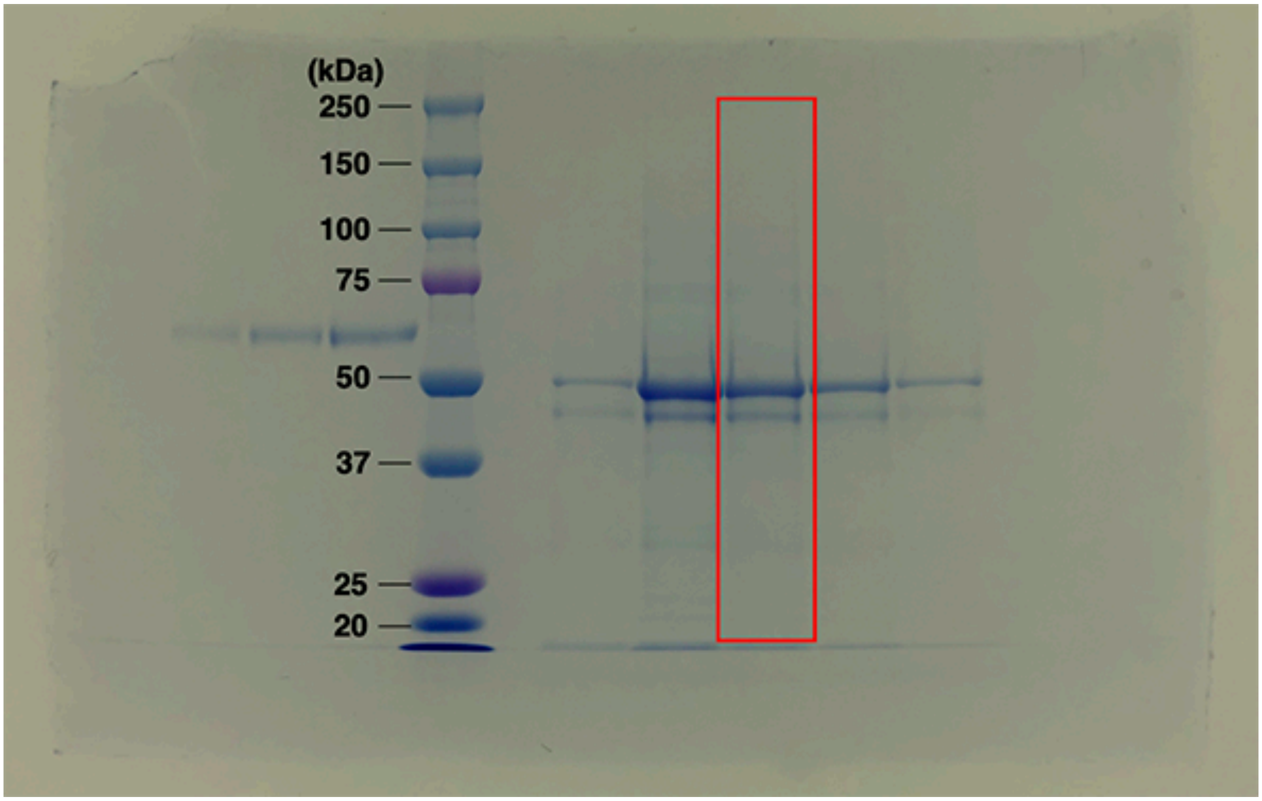

Figure S4: Full-length image of the gel. The red square indicates the cropped area shown in Figure S3.
